# Bridging the Gap: Harnessing Plant Bioactive Molecules to Target Gut Microbiome Dysfunctions in Amyotrophic Lateral Sclerosis

**DOI:** 10.3390/cimb46050271

**Published:** 2024-05-08

**Authors:** Mirela Pribac, Anca Motataianu, Sebastian Andone, Elena Mardale, Sebastian Nemeth

**Affiliations:** 1 Doctoral School of Biomedical Sciences, University of Oradea, 410087 Oradea, Romania; pribac.mirelaioana@student.uoradea.ro (M.P.); snemeth@uoradea.ro (S.N.); 2 Ist Neurology Clinic, Mures County Clinical Emergency Hospital, 540136 Targu Mures, Romania; sebastian.andone@umfst.ro; 3 Department of Neurology, University of Medicine, Pharmacy, Science and Technology, 540142 Targu Mures, Romania; 4 Independent Researcher, 500091 Brasov, Romania; elena.mardale@gmail.com

**Keywords:** gut microbiome, amyotrophic lateral sclerosis, plant molecules, essential oils, oxidative stress, neurodegenerative disease

## Abstract

The correlation between neurodegenerative diseases and the gut microbiome is increasingly evident, with amyotrophic lateral sclerosis (ALS) being particularly notable for its severity and lack of therapeutic options. The gut microbiota, implicated in the pathogenesis and development of ALS, plays a crucial role in the disease. Bioactive plant molecules, specifically volatile compounds in essential oils, offer a promising therapeutic avenue due to their anti-inflammatory properties and gut-modulating effects. Our narrative review aimed to identify microbiota-associated bacteria in ALS and analyze the benefits of administering bioactive plant molecules as much-needed therapeutic options in the management of this disease. A comprehensive search of PubMed database articles published before December 2023, encompassing research on cell, human, and animal ALS models, was conducted. After selecting, analyzing, and discussing key articles, bacteria linked to ALS pathogenesis and physiopathology were identified. Notably, positively highlighted bacteria included *Akkermansia muciniphila* (Verrucomicrobia phylum), *Faecalibacterium prausnitzii,* and *Butyrivibrio* spp. (Firmicutes phylum). Conversely, members of the *Escherichia coli* spp. (Proteobacteria phylum) and *Ruminococcus* spp. (Firmicutes phylum) stood out negatively in respect to ALS development. These bacteria were associated with molecular changes linked to ALS pathogenesis and evolution. Bioactive plant molecules can be directly associated with improvements in the microbiome, due to their role in reducing inflammation and oxidative stress, emerging as one of the most promising natural agents for enriching present-day ALS treatments.

## 1. Introduction

Amyotrophic lateral sclerosis (ALS) is a heterogenous neurodegenerative disorder primarily affecting the motor neurons (MNs) from the motor cortex and spinal cord. Despite being recognized for over a century, since Charcot’s initial description in 1869, ALS remains a challenging condition due to both its complex etiopathogenic mechanisms and the lack of therapeutic prospects. Only 5–10% of cases manifest as familial ALS, stemming from inheritable genetic mutations, including those within genes such as chromosome 9 open reading frame 72 (C9orf72), superoxide dismutase 1 (SOD1), fused in sarcoma gen (FUS), and TAR DNA Binding Protein (TARDBP) [1,2].

Characterized by a lifetime risk of one in 300 and a typical onset age of around 60 years, ALS carries a mean survival time of two to five years, rendering it among the most devastating neurodegenerative disorders. Annually, approximately 30,000 deaths worldwide are attributed to ALS, influenced by a combination of environmental, genetic, and potentially stochastic factors [3].

The pathogenesis of ALS is a complex phenomenon characterized by interconnected and overlapping pathways, including oxidative stress (OS), mitochondrial dysfunction, abnormal RNA processing, neuroinflammation, glutamate excitotoxicity, and environmental influences. All these factors are believed to play contributory roles [4,5].

Viral genes, such as endogenous retrovirus-K, have been linked to ALS neuropathology due to their ability to interact with neuronal pathways via both cell-associated and cell-free mechanisms [6]. New findings indicate the potential impact of a conotoxin-like protein within the ERVK transcription genes with a more specific potential role in ALS neuropathology through its impact on inflammation, immune gene expression, and motor neuron degeneration [7]. However, given the complex viral genomic mutations, more studies are needed to investigate the gut virome epigenetic regulation of brain disorders.

A hallmark feature of neurodegenerative processes is the dysfunction of the inflammatory response. Both neuroinflammation and OS have been identified as pivotal aspects that must be considered in understanding the initiation and progression of neurodegenerative disorders, as they are intricately interconnected in their pathogenesis [8,9,10].

The gut microbiota are now recognized as a critical contributor to maintaining host health, impacting various physiological functions such as circadian rhythm, metabolism, and immunity [11,12]. Notably, the connection between the intestinal microbiota and the brain, forming the gut–brain axis (GBA), is of particular significance. This axis involves complex networks, including the central nervous system (CNS), the autonomic nervous system, the enteric nervous system, and the hypothalamic–pituitary–adrenal axis, all of which are crucial for ALS progression [13,14]. The GBA functions as a bidirectional communication system, with the autonomic and enteric nervous systems regulating gastrointestinal function. 

Mounting evidence suggests that the gut microbiota influence the CNS via neuroimmune and neuroendocrine pathways, mediated by microbial-derived molecules. Gut microbiota disturbances linked to pathological conditions can trigger pro-inflammatory cytokine production, leading to neuroinflammation. Moreover, gut microbiota dysfunction disrupts intestinal permeability, allowing toxic substances and inflammatory mediators to enter the circulatory system, affecting the onset and progression of ALS. Therefore, manipulating the microbiota to regulate the production of bacterial metabolites such as short-chain fatty acids (SCFAs), secondary bile acids, gamma-aminobutyric acid, or serotonin, emerges as a crucial strategy in ALS inflammation management [15,16]. 

Serotonin, a microbial metabolite, heavily depends on dietary tryptophan for its synthesis, which takes place mainly in the gut [17]. Research has found that treating murine microglial cells and primary microglia cultures with serotonin heightens exosome-associated proteins and insulin-degrading enzyme. Specific serotonin receptor antagonists impede this effect [18]. Exosomes have emerged from recent studies as a type of extracellular vesicle that, importantly, mediates intercellular communication as well as other physiological mechanisms such as cytokine secretion [19]. Given these mechanisms, serotonin likely plays a pivotal role in cytokine-carrying exome release from microglia [18]. Hence, serotonin-induced microglial exosomes could significantly impact neuroinflammation and associated disorders such as ALS [19].

Disease-modifying drugs like riluzole and edaravone are designed to attenuate progression, yet their efficacy remains constrained [20]. This underscores the necessity to explore, investigate, and integrate novel and efficient approaches that could optimize both similar and complementary pharmacokinetics, effectively targeting the primary pathological mechanisms in ALS.

The modulation of the GBA in ALS through natural therapies shows promise as a valuable tool for understanding and managing the disease [21,22]. In recent years, there has been a notable shift towards exploring complementary and plant medicine for managing neurodegenerative diseases like ALS, with a particular focus on elucidating the mechanisms involved in neurodegeneration and microbiome modulation [23]. Essential oils (EOs), derived from various plant sources, have emerged as promising candidates, given their rich bioactive compounds and recognized effects on neuroinflammation and gut microbiota.

Essential oils have undergone extensive investigation for their antioxidant and anti-inflammatory properties, essential in combating neuroinflammation, a hallmark of neurodegeneration. Bioactive plant molecules (BPMs) like linalool, limonene, benzyl benzoate, and benzyl alcohol found in EOs have demonstrated anxiolytic and antidepressant effects, acting through interactions with the GABAergic pathway [24,25]. Other BPMs, such as (E)-cinnamaldehyde predominantly found in cinnamon EO and eugenol from clove EO, have shown a significant reduction in neuroinflammation by inhibiting the release of pro-inflammatory cytokines [26,27]. Moreover, oral supplementation with Wild Orange (*Citrus sinensis*) EO has been observed to enhance the growth of beneficial bacterial species such as *Bifidobacterium* spp. in the colon, leading to modifications in the composition and function of the intestinal microbiome [28]. This modulation of the gut microbiota is crucial as it impacts various metabolic pathways, including amino acid, carbohydrate, and lipid metabolism, which play significant roles in neurodegenerative processes [29].

**Figure 1:**
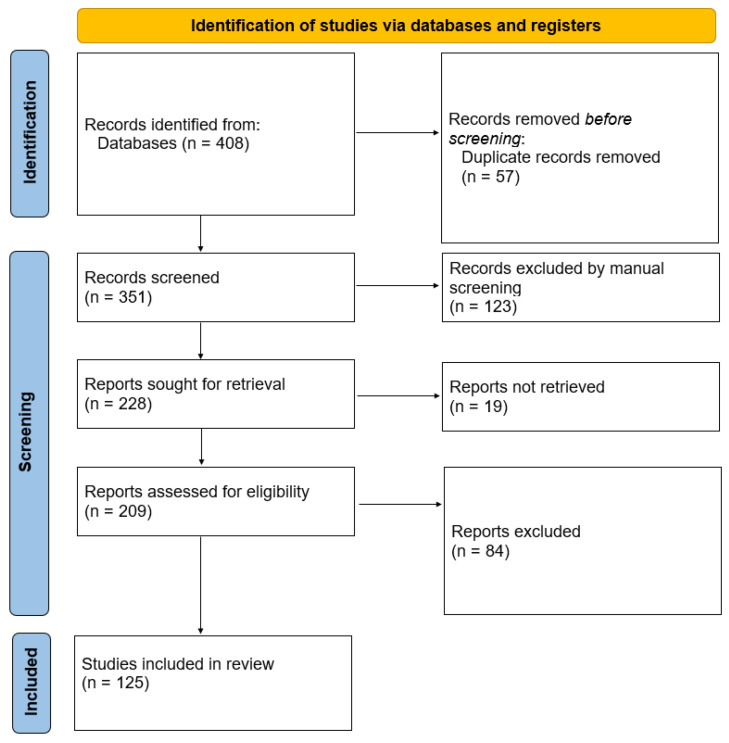
Flow diagram for the review.

Our narrative review aimed to identify microbiome-associated bacteria in ALS and evaluate the potential benefits of BPMs across the mechanisms implicated in the disease. We conducted a comprehensive search of PubMed, focusing on relevant articles published before December 2023, using key terms such as microbiota, essential oils, and ALS. The selected papers underwent meticulous analysis following the guidelines outlined in Figure 1.

## 2. The Microbiome and Amyotrophic Lateral Sclerosis

### 2.1. The Healthy Microbiome

The gastrointestinal tract, home to multiple trillions of microorganisms forming the gut microbiota, is influenced by host genetics and environmental exposure [13]. The gut microbiome, encompassing bacteria, fungi, viruses, protozoa, and parasites, plays a critical role in influencing systemic health and the development of disease [30].

Alterations in the gut microbiota, associated with changes in the mediation of immunity, affect normal physiological functions, influencing the onset of disease. Pathological states, conversely, impact the gut, determining changes in the microbiota. The gut microbiota, often referred to as the human “second genome”, contain a vast repertoire of 3.3 million genes, surpassing the human genome by more than an order of magnitude. Their composition has been revealed through next-generation sequencing, indicating their connection to various systemic diseases [31]. The number of bacteria in the human gut is nearly equivalent to the number of human cells, significantly expanding its metabolic capabilities, comparable to the functions of the liver [32].

Comprising four major phyla (Firmicutes, Bacteroidetes, Proteobacteria, and Actinobacteria) and two minor phyla (Fusobacteria and Verrucomicrobia), the human gut microbiota see Firmicutes and Bacteroidetes representing around 90% [33,34].

The density and composition of microbes throughout the gastrointestinal (GI) tract are affected by chemicals, dietary components, and immunological elements, alongside variations in pH levels and transit times. While the stomach and small intestine restrict microbial proliferation, the colon, marked by anaerobic conditions, slower food transit, water absorption, and food fermentation, harbors a dense microbial population [35].

Gut microbiota communities exhibit dynamism, adapting to both internal and external influences. However, despite individual differences, the notion of a “core microbiota” implies the stable presence of predominant organisms at a functional level [36]. Variations in this “core microbiota” mark the deviations between eubiosis and dysbiosis, between what is known, to date, to be a balanced versus an unbalanced intestinal milieu [37]. 

The gut microbiota, closely intertwined with human well-being, contribute to various physiological functions including the immune response, digestive processes, metabolism, and the transmission of neurological signals [38]. The Firmicutes/Bacteroidetes ratio is regarded as a marker of gut microbiota health, providing valuable information on disease susceptibility and potential treatment strategies [39].

Elevated Firmicutes and decreased Bacteroidetes levels have been associated with a range of health issues, such as type 2 diabetes, obesity, and dementia [40,41]. A “healthy” microbiota deficiency or dysbiosis, characterized by alterations in the gut microbiome composition and function, is linked to metabolic and gastrointestinal diseases, renal disease, and neurodegenerative diseases [38] including ALS. 

Recent studies have shown that gut Firmicutes play a key role in fermenting dietary fiber and interacting with the intestinal mucosa, contributing to overall health. Promoting a healthy abundance of this phylum could offer insights into probiotic therapy and dietary interventions, with potential benefits for health and for the management of incurable diseases such as ALS. Specifically, increasing the abundance of Firmicutes like *Faecalibacterium prausnitzii* spp., *Lactobacillus* spp., and *Roseburia* spp. may hold promise for promoting health [42].

Bacteroidetes and Proteobacteria, comprising some of the main lipopolysaccharide (LPS) bacteria, such as the Bacteroidetes member *Bacteroides fragilis* spp. (*B. fragilis*) and Proteobacteria member *Escherichia coli* spp. (*E. coli*), are the most abundant Gram-negative bacteria in the middle and lower colon of the human GI tract. Under optimal bacterial growth conditions, the abundance of *B. fragilis* spp. or *E. coli* spp. doubles approximately every 20 min and can have a lifespan of up to several hundred minutes, unless specific biophysical processes such as dormancy or hibernation are in operation. Consequently, large quantities of neurotoxins generated by *B. fragilis* spp. or *E. coli* spp. can accumulate rapidly [43] and this is precisely one area where the microbiome elicits pro-inflammatory effects on the host, though various mechanisms capable of inducing inflammatory signaling in neuronal–glial cells and play a role in the activation of the NF-κB pro-inflammatory transcription factor complex [44]. Moreover, Gram-negative bacterial lipopolysaccharide (LPS), abundant in the outer membrane of bacteria, is pivotal in host–microbial interactions. During microbial overgrowth and infection in the gut and other anatomic sites, LPS alterations occur, aiding adhesion, colonization, and immune evasion. Therefore, the control of LPS-positive bacteria in the gut is one of the foremost strategies in balancing the microbiome and preventing complications in most chronic and degenerative conditions such as ALS.

The gut microbiome exerts a profound influence on the host through the production of metabolites, with primary metabolites playing essential roles in cellular functions and energy metabolism. Short-chain fatty acids (SCFAs), mainly represented by butyrate, acetate, and propionate, are byproducts of fermentation and act as energy sources systemically or locally for various tissues. Additionally, SCFAs participate in gene regulation and T-cell modulation, and impact the brain. Secondary metabolites, resulting from enzymatic modifications of primary metabolites, are crucial mediators in gut–microbiome interactions. The bacterial conversion of tryptophan into indole or indole derivatives and the generation of the neurotransmitter γ-aminobutyric acid (GABA) from glutamate highlight the diverse roles of secondary metabolites. Vitamins, essential, but not synthesized by the host, exhibit variability in their dietary sources, microbial synthesis, and host requirements, presenting a fascinating area of investigation into host–microbe relationships. This intricate interplay underscores the dynamic and multifaceted nature of the gut microbiome and its impact on host health [45].

### 2.2. Microbiome Alterations in ALS

Initial studies of the microbiome in ALS consistently found dysbiosis to be present, both through potentially harmful bacterial overgrowth and through the reduced biodiversity of potentially beneficial bacterial strains. These studies proposed that pro-inflammatory dysbiosis might stem from microbial imbalance, compromising the intestinal epithelial barrier and triggering immune/inflammatory responses, thus playing a role in ALS pathogenesis [46]. Patients with ALS who exhibit heightened richness and diversity in their microbiome, along with a higher Firmicutes to Bacteroidetes ratio, are noted to face a heightened risk of premature mortality [47], this association being observed at a later stage in disease progression. However, one study found no significant alteration in the composition of gut microbiota among ALS patients [48].

Since one main concern in the microbiota of ALS regards inflammation, certain researchers have investigated how alterations in the microbiota can influence the phenotype attenuating inflammation, even in the presence of a mutation in C9ORF72, the most prevalent genetic variant associated with ALS [49].

An important prospective longitudinal study investigating the composition of the gut microbiota in ALS revealed significant differences in the gut microbiome of ALS patients during disease progression compared to controls, regardless of the disability level. The microbiome pattern changes as the disease advances. Compared to healthy individuals, the microbiome of ALS patients displays a notable shift in the relative abundance of bacterial communities, with a decrease in potentially protective bacteria and increase in neurotoxic bacteria [50]. These results suggest that addressing the microbiome could become a fundamental tool in the pathogenesis and management of ALS. 

One study indicates that the levels of butyrate-producing bacteria are lower in the microbiome of ALS patients during disease progression [51].

Another study found ALS-associated dysbiosis was directly correlated with high inflammatory markers such as calprotectin, fecal secretory IgA, and eosinophilic protein X, but, on the contrary, was also correlated with high counts of glutamate-producing bacteria such as *Lactobacillus* spp., *Bifidobacterium* spp., and *Odoribacter* spp. [52]. These findings suggest that alterations in the microbiota may modulate the clinical course of the disease rather than serving as a risk factor for its onset.

An animal study revealed pre-symptomatic dysbiosis and altered metabolite profiles in the microbiome of ALS patients. The experiments unveiled that *Akkermansia muciniphila* (*A. muciniphila*), beneficial bacteria for the gut immunity and lining, was found to be consistently decreased in the microbiome of ALS patients, whereas *Ruminococcus torques* and *Parabacteroides distasonis* spp. worsen ALS symptoms [53]. One study found low *Ruminococcus* spp. to be possibly responsible for the low Firmicute/Bacteroidetes ratio in ALS patients as observed during the progression of the disease [52], but these findings could only be interpreted as detrimental if we consider the butyrate synthesis potential of some species such as *R. torques*, which is essential for the balance of the microbiome generally. But the exact role of the Ruminococcus genus is yet unclear, and this is because other species, such as *R. gnavus,* have been linked to various gastrointestinal diseases [54].

Another promising perspective, still under hypothesis, is the possibility proposed by a group of researchers for a potential motor neuron toxin that could be produced by *Clostridium* spp. in the gut, leading to the onset of ALS under certain conditions. Since certain neurotoxins, including tetanus and botulinum toxins, are produced by *Clostridium* spp. found not only in soil but also in human/animal guts, the hypothesis arises that they could potentially target motor systems, with diverse clinical effects [55].

## 3. Essential Oils and ALS

### 3.1. General Overview of Bioactive Plant Molecules from Essential Oils

Plants produce two types of oils: fixed oils, comprising glycerol esters bonded to three fatty acids (known as triacylglycerols or triglycerides), and essential oils (EOs). EOs, also termed volatile oils, are intricate natural blends of volatile, lipophilic substances that are structural and functional constituents of aromatic plants. Most essential oils are colorless, liquid at room temperature, with a lower density than water. Additionally, essential oil components have a low molecular weight, with some being optically active, and they dissolve in many organic solvents (such as ether, alcohol, acetone) while remaining insoluble in water [56].

Typically, EOs comprise roughly 20–60 molecular components at varying concentrations, with some containing hundreds of different substances. Generally, two or three molecules typically dominate, making up 20–70% of the oil. For instance, 1,8-cineole or eucalyptol accounts for 70–90% of *Eucalyptus globulus Labill.* EO [57], thymol constitutes 37–55% of thyme leaf (*Thymus* spp.) EO [58] and cinnamaldehyde makes up 60–90% of cinnamon bark and leaf (*Cinnamomum zeylanicum*) EO [59]. These major components often determine the biological properties of EOs, although minor molecules can also influence their bioactivity, either enhancing or counteracting the effects of major components.

Essential oil compounds fall into two primary groups: terpenoids (the predominant group) and non-terpenoids, represented mainly by phenylpropanoids. These compounds are hydrocarbons or oxygenated derivatives, belonging to various chemical classes such as alcohols, aldehydes, oxides, ketones, esters, amides, phenols, amines, nitrogen, and sulfur compounds [60].

Most EOs and their main bioactive molecules, such as carvacrol, limonene, citral, eugenol, and cinnamaldehyde, have been studied for their antibacterial, antiviral, and antifungal effects, both in vivo and in vitro [61,62,63].

Numerous studies have documented the cytotoxic and the antitumoral effects of EOs with a wide range of proposed mechanisms. These include the induction of apoptotic cell death, linked to elevated levels of ROS, the activation of the MAPK pathway, and the inhibition of the NF-κB and AKT/mTOR pathways. For instance, *Zataria multiflora Boiss* EO stimulated ROS production and apoptosis in colon cancer cells [64]. Similarly, garlic (*Allium sativum*) BPMs prompted cytotoxicity in leukemia cells by enhancing ROS levels at that specific tumor site and triggered apoptosis [65]. Furthermore, Litsea seed (*Litsea cubeba*) EO induced apoptotic cell death by inhibiting the AKT/mTOR pathway [66].

More studies are arising for the promising antioxidant molecules of EOs. Given that the composition of EOs can be affected by numerous factors, understanding the effectiveness and mechanisms of action of each component enables us to anticipate the activity of the specific oil [67]. BPMs can be categorized into three main groups based on their chemical mechanism of peroxidation inhibition: phenols, serving as agents with radical trapping activity; highly oxidizable compounds, assisting in termination; and 1,4-cyclohexadienes, capable of generating the reducing HOO radicals [68].

### 3.2. The Interplay of Bioactive Plant Molecules and the Microbiome in ALS

Essential oils exhibit diverse biological activities through the multitude of molecules they comprise, including antioxidant, anti-inflammatory, and antiproliferative properties [69]. The undebatable effects of BPMs on bacteria indicate that they could potentially modulate the microbiome, influencing microbial diversity and composition, further impacting overall health in various pathologies including ALS. It is interesting to observe the promising role of various BPMs in influencing specific bacteria that characterize the microbiota of ALS, as potential tools for complementing existing medication and therapeutic approaches in this challenging disease. 

At the genus level, the bacteria most studied in the microbiome of ALS include *Faecalibacterium prausnitzii*, *A. muciniphila*, *Dorea* spp., *Clostridium* spp., *Ruminococcus* spp., *Escherichia Coli* spp., and *Prevotella* spp. 

Although there is no absolute consensus on the precise alterations in the microbiome of ALS patients, due to both the scarcity and the limitations of existing research, most authors report similar findings.

At phylum level, there seems to be a fair amount of converging results pointing to the lower levels of Firmicutes and Verrucomicrobia [51,70,71], elevated levels of Bacteroidetes and Proteobacteria [70,71,72,73] with only two studies indicating the reduction in Actinobacteria [71] in ALS. These alterations represent a consensus since most studies have quantified the phyla per ensemble, whereas at a more detailed analysis of species, some bacteria pertaining to Firmicutes phylum are increased (*Ruminococcus* spp., *Clostridia, Enterococcus* spp.), despite the general decrease in the entire phylum (main species decreased *Faecalibacterium prausnitzii*, *Roseburia intestinalis, Lactobacillus* spp.). Similarly certain bacteria pertaining to Bacteroidetes phylum (*Prevotella* spp.) are decreased despite the overall increase in the phylum. 

One study found an increase in bacterial species belonging to *Clostridium* spp. and Ruminococcaceae in ALS patients, the latter known to play a critical role in transforming primary bile acids (PBAs) to secondary bile acids (SBAs). As PBAs transit from the small intestine to the colon, they undergo conversion to SBAs through microbial biotransformation, with the key step being the 7α-dehydroxylation reaction. In the colon, nearly all cholic acid (CA) and chenodeoxycholic acid (CDCA) are converted to deoxycholic acid (DCA) and lithocholic acid (LCA), respectively. However, only a few bacteria, primarily pertaining to Ruminococcaceae (family) and the Clostridium genus, can perform 7α-dehydroxylation. Consequently, as *Ruminococcus* spp. proliferates, the SBA concentration increases, highlighting the intricate interplay between altered gut microbial communities and bile acid metabolism in ALS patients and its potential implications for cognitive decline [72].

There seems to be fair consensus concerning the high level of some *Ruminococcus* spp. in neurodegenerative diseases [74]. High levels of *Ruminococcus torques* and *Parabacteroides distasonis* have been associated with the worsening of ALS symptoms [47]. Therefore, solutions such as BPMs from tea tree EO, demonstrated to have a relevant inhibitory capacity of *Ruminococcus* spp. [75], are promising for the management of ALS. 

Human *Clostridium* spp. are significantly reduced by thymol, methyl isoeugenol, eugenol, geraniol [76], nerolidol, and thymol, respectively [77].

Other authors found that ALS patients exhibited significantly higher concentrations of *Escherichia coli* (*E. coli*) compared to controls [78], along with elevated levels of the Enterobacteriaceae family. EO supplementation in experimental broilers reduced the populations of cecal *E. coli*, a recognized pathogen and LPS-producing bacterium responsible for the production of ROS in ALS. Similarly, among many similar studies, an in vitro study demonstrated that carvacrol and thymol could inhibit the growth of *E. coli* [79], making these BPMs strong candidates for lowering the level of this bacterium usually found in high concentration in the gut of ALS patients. 

On the contrary, another study identified a greater percentage of the Actinobacteria and Verrucomicrobia phyla, as well as the Cyanobacteria phylum in ALS subjects. The patients studied showed an increased abundance of *Lactobacillus* spp., *Citrobacter* spp., and *Coprococcus* spp. [50]. Conversely, another study identified a reduced *Bifidobacterium pseudocatenulatum* abundance, a potentially helpful species pertaining to the Actinobacteria phylum [53].

Additionally, genera belonging to Enterobacteriaceae family (such as *E. coli* spp. and *Shigella* spp.), including *A. muciniphila*, *Eubacterium eligens*, Odoribacter spp., *Bifidobacterium* spp., *Pseudoflavonifractor* spp., as well as other genera within the Prevotellaceae family, were found to be more abundant in ALS patients [50], compared to other studies that show a decrease in helpful *A. muciniphila*, *Faecalibacterium prausnitzii*, and *Prevotella* spp. Out of these, the overgrowth of Enterobacteriaceae and of *Citrobacter* spp. specifically is potentially problematic due to the possibility of causing opportunistic infections such as urinary tract infections and gastroenteritis. Moreover, there is a fair amount of microbiota-derived oxidative stress and inflammation signaling mediators with the overgrowth of potentially pathogenic bacterial strains. The strong potential of BPMs such as carvacrol and thymol to directly decrease the bacteria from the Enterobacteriaceae family [80] and to reduce oxidative stress has been studied extensively. BPMs from sage EO (*Salvia officinalis*) such as α-thujone, camphor, 1,8-cineole, and β-thujone exhibit promising antibacterial activity against *Enterococcus faecalis* and *Citrobacter freundii* and could potentially be used therapeutically in ALS to balance this bacterial overgrowth [81].

An animal study revealed pre-symptomatic dysbiosis and altered metabolite profiles in the microbiome of ALS. The results unveiled that *Akkermansia muciniphila* yields a beneficial effect [53]. *A. muciniphila* directly binds to enterocytes, enabling colonization and providing energy to colonocytes. Its degradation of mucin stimulates mucin production, increasing mucin thickness and strengthening epithelial integrity, therefore acting as one of the most important bacteria of the good “core microbiota” [82]. Studies found that these bacteria ameliorate the general health status of ALS in mice, possibly through nicotinamide production [83]. 

A substantial reduction in the levels of the beneficial bacterium *Faecalibacterium prausnitzii* was observed in the fecal samples of ALS patients compared to healthy adults [84]. Typically, this genus maintains a level above 5% in the gut of healthy individuals, and a decrease in *Faecalibacterium prausnitzii* levels is often associated with the development of conditions such as coeliac disease and irritable bowel syndrome [85]. 

Bitter orange (*Citrus aurantium* var. *amara*) EO exhibits a concentration-dependent inhibition of IL-10 and TNF-α cytokine production when exposed to pro-inflammatory stimuli from LPS-positive bacteria. This reduction in the pro-inflammatory environment may support the growth of beneficial probiotic strains, including *A. muciniphila* and *Faecalibacterium prausnitzii*, with a potential to ameliorate inflammatory bowel disease [86]. Therefore, the main BPM of this EO, the monoterpene limonene, could be associated with the increase in bacteria that are essential in gut protection via the integrity of the mucus lining, generally decreased in ALS patients. 

Alterations in butyrate metabolism and the deficiency in *Prevotella* spp. could hold significance for ALS treatment, due to the role of *Prevotella* spp. in synthesizing folate and thiamine [87]. Oral supplementation with a combination of BPMs such as 10.0% cinnamaldehyde and 5.0% thymol in an in vivo animal model was shown to increase the level of *Prevotella* spp. in the gut microbial structure, with positive effects on the overall health parameters of the host [88].

*Methanobrevibacter* spp. overgrowth (bacteria pertaining to the Euryarchaeota phylum) was identified in the microbiome of ALS patients [84]. Previous studies have noted that *Methanobrevibacter* spp. utilizes short-chain fatty acids (SCFAs) as a substrate to produce CH4, and an increase in the abundance of this genus is associated with a potential decline in host weight [89]. Additionally, research on the archaeal composition distribution revealed that the relative abundance of methanogens in patients with irritable bowel syndrome was significantly higher than that in healthy individuals [90]. Similar results were observed in the fecal samples of ALS patients compared to those from healthy individuals. Therefore, the dietary administration of EOs could be a strategy to lower methanogenic archaea, and one study found that a combination of ajwain (*Trachyspermum copticum*) EO, lemongrass (*Cymbopogon citratus*) EO, and clove (*Syzygium aromaticum*) EO blended in equal parts has a significant effect on *Methanobrevibacter* spp. [91].

Contrary to common consensus in ALS, some studies found an increase in the Firmicutes phylum, but found *Dorea* spp. to be the common denominator, at a genus level. *Dorea* spp., pertaining to the Clostridia class, with its major end products of glucose metabolism being ethanol, is considered a potentially harmful bacteria, therefore being negatively associated with the microbiome of ALS patients [92]. Other studies have identified elevated levels of some other Clostridia, such as *Oscillibacter* spp., *Anaerostipes* spp. and the Lachnospiraceae family, in the microbiome of ALS patients [51,52]. As previously mentioned, some BPMs such as linalyl acetate from lavender (*Lavandula angustifolia*) EO or carvacrol from oregano (*Origanum vulgare*) EO and winter savory (*Satureja montana*) EO, strongly reduce the Clostridia count [93], and therefore could be considered valid molecules to control *Dorea* spp. overgrowth in ALS microbiota.

A reduction in the level of antimicrobial α-defensin 5 was found in the gut composition of ALS, correlated with a reduction in the abundance of *Butyrivibrio fibrisolvens*, a butyrate-producing bacterium with a regulatory role in the intestinal epithelium and its potential permeability through mechanisms involving the inflammatory cytokine IL-17 [94]. Low levels of eight other dominant butyrate producers, including *Eubacterium rectale* and *Roseburia intestinalis* to name a few, have been found in patients with ALS, these findings indicating a reduced immune-modulating gut flora [51].

Beyond its role as a main butyrate producer, *Butyrvibrio fibrisolvens* could be considered a therapeutic probiotic in managing digestion-related disorders but also tumoral formation, due to its ability to increase the number of NK and NKT cells, and immunoglobulin A in the gut [95]. An animal study demonstrated the potential of a standardized solution of three BPMs, cinnamaldehyde, thymol, and eugenol, to support the abundance of *Butyrivibrio fibrisolvens*, therefore setting the ground for its same application in human health [96].

Overall, the dysregulation of the gut microbial composition in ALS patients, characterized by the proliferation of potential pathogens and the reduction in probiotic organisms, potentially impacts the production of nitric oxide (NO), gamma-aminobutyric acid (GABA), and SCFAs [97]. Consequently, this dysbiosis could also be associated with the pathogenesis of ALS, exacerbating the imbalance in the intestinal microbiota and perpetuating a detrimental cycle for host health. These findings lay the groundwork for understanding the pivotal role of specific bacteria in ALS onset and suggest that targeting the inhibition of potential pathogen growth and the enhancement of probiotic strains through complementary strategies, such as the use of natural BPMs from essential oils, could aid in the treatment of ALS [73].

### 3.3. Microbiome-Associated Dysfunctions in ALS 

Gastrointestinal motility disorders, like delayed gastric emptying and constipation, significantly affect patients with ALS [98]. Dysbiosis may contribute to these dysfunctions through interactions with the enteric nervous and immune systems. Understanding this link is crucial for developing targeted interventions to restore gut balance and improve clinical outcomes.

Gastroparesis, frequent in ALS, results from enteric neuron dysfunction due to neurodegeneration, disrupting peristalsis. This leads to delayed food passage, causing symptoms like early satiety, bloating, nausea, and vomiting [99], contributing to sarcopenia.

Plant therapies and BPMs from EO show promise in ameliorating delayed gastric emptying, enriching conventional treatments with natural solutions. Emerging research has highlighted the therapeutic potential of molecules such as anethole, a major BPM of fennel (*Foeniculum vulgare*) EO, which has garnered attention for its therapeutic potential for delayed gastric emptying [100]. Menthol, the BPM in peppermint (*Menta piperita*) EO significantly improved gastric emptying via in vivo administration [101]. 

Constipation, an organic dysfunction common to most neurodegenerative conditions, worsens due to altered colonic motility and reduced physical activity and fluid intake, common in ALS patients. Plant BPMs like menthol and thymol offer promising solutions to manage constipation [102]. Research substantially supports the notion that BPMs derived from EOs possess therapeutic properties that may offer promising avenues for the development of natural solutions for gastrointestinal organic disorders in both primary GI diseases and in secondary ones, such as organic gut dysfunction in ALS.

## 4. The Potential of Bioactive Plant Molecules to Regulate Microbiome-Derived Oxidative Stress and Inflammation in ALS

### 4.1. Oxidative Stress

Amyotrophic lateral sclerosis involves various cellular and molecular processes, where oxidative stress plays a major role in the development of the disease. Oxidative stress (OS), primarily stemming from mitochondrial dysfunction, exacerbates ALS pathology. Mutations in ALS-related genes and environmental factors contribute to OS, necessitating further research for targeted therapeutic development [10].

The phenolic components of EO, including carvacrol, thymol, and eugenol to name a few, are known for their oxidative stress-fighting activity that arises from phenolic compounds donating the phenolic hydrogen atom to ROO radicals, generating resonance-stabilized phenoxyl radicals that halt the oxidative chain reaction [103].

In an induced neurotoxicity animal model, researchers investigated the effects of pine (*Pinus halepensis*) EO with caryophyllene, phenyl isovalerate, β-myrcene, and α-pinene as its main BPMs and found inhibitory effects on acetylcholinesterase activity in the hippocampus, potentially reducing Aβ aggregate formation. Additionally, this EO demonstrated potent antioxidant properties, enhancing the activity of antioxidant enzymes, namely, superoxide dismutase, catalase, and glutathione peroxidase, while increasing glutathione levels [104]. These effects collectively countered oxidative stress and ameliorated neurological status. In other models of neurotoxicity induced by titanium dioxide nanoparticles or aluminum, limonene, a major BPM in the composition of bergamot (*Citrus bergamia*) EO, was studied. Limonene was able to alleviate oxidative damage and neuroinflammation in the hippocampus and frontal cortex regions, increasing the levels of antioxidant enzymes and decreasing the release of pro-inflammatory cytokines (TNF-α, IL-1β, IL-6), lipid peroxidation, and DNA damage [105,106]. Carvacrol, thymol, eucalyptol, linalool, linalyl acetate, and asarone are all BPMs present in EOs that have been demonstrated to reduce the expression levels of pro-inflammatory mediators in neuroinflammation and have the potential for action in neurodegeneration [107,108,109,110]. 

Other BPMs, such as alpha-zingiberene, ar-curcumene, and a-sesquiphellandrene from ginger (*Zingiber officinale*) EO administered orally for one month, demonstrated antioxidant properties by enhancing glutathione levels in an in vivo study [111].

Bioactive plant molecules seem to be efficient in multiple OS-related mechanisms, making them promising antioxidant tools with the potential to act either synergically or complementarily to present medical treatments for ALS.

### 4.2. Inflammation

Beyond nutrient metabolism, gut microorganisms interact with the immune system in ways that either promote or prevent inflammation. Some microbes are associated with anti-inflammatory mechanisms, stimulating regulatory immune cells to inhibit inflammation [112]. Conversely, bacteria can regulate intestinal permeability, with certain species promoting a “leaky gut”, where microbial metabolites enter the bloodstream. In response, the body initiates an inflammatory response by producing cytokines and other mediators [113]. Additionally, cells within the gut’s epithelial tissue deliver bacterial metabolites to immune cells, fostering inflammation at both local and systemic levels. Such persistent mechanisms may lead to subacute or chronic inflammation, potentially driving the development of diseases like inflammatory bowel disease, diabetes, or cardiovascular disease.

In exploring the intricate connections between the gut microbiota and inflammatory markers, several key mechanisms have been proposed. Lipopolysaccharides (LPS), prevalent in Gram-negative bacteria, increase in conditions like obesity, causing local and systemic inflammation when the gut barrier is disrupted [114,115]. LPS can compromise the permeability of the intestinal wall, allowing neurotoxins to enter the bloodstream, triggering the release of pro-inflammatory cytokines, activating the Toll-like receptor (TLR4) and nuclear factor kappa B (NF-κB) pathway [92]. Pro-inflammatory cytokines cross the blood–brain barrier in ALS, fueled by increased Enterobacteriaceae and subsequent LPS production. Short-chain fatty acids (SCFAs), products of microbial carbohydrate metabolism, play a pivotal role, with butyrate exhibiting anti-inflammatory effects and acetate showing variable impacts on insulin resistance and weight [116]. Bile acids, influenced by the activity of gut bacteria, have the potential to contribute to inflammation and obesity by engaging the farnesoid X receptor signaling pathway in both adipocytes and enterocytes [117]. C-reactive protein, identified as an acute-phase reactant linked to metabolic syndrome and cardiovascular disease, experiences elevated levels in circulation when interleukin-6 (IL-6) is released by macrophages and T cells [118]. 

In an animal microbiome model, obese mice exhibited a diminished proportion of *A. muciniphila* alongside increased plasma CRP levels. However, the study does not elucidate whether CRP influences the gut microbiota or if the gut microorganisms contribute to its escalation [119]. The presence of *Phascolarctobacterium*, a genus associated with lower CRP levels, produces propionate, an anti-inflammatory short-chain fatty acid that inhibits pro-inflammatory cascades by suppressing nuclear factor kappa-B (NFκB) activity [120,121]. Similarly, *Faecalibacterium prausnitzii* shows an inverse correlation with CRP levels [122], indicating that CRP, as a downstream inflammatory marker, can be regulated by the anti-inflammatory metabolic products of specific gut microbes. The evaluation of the baseline serum and microbiota data in healthy individuals with a BMI over 25 revealed that higher CRP levels were associated with a lower abundance of Lactobacillus and Bifidobacterium but a higher abundance of *E. coli* spp. and Bacteroides [123]. Cytokines, including TNF-α and IL-6, are implicated in inflammation and metabolic disorders, correlating with specific gut microbial species [124]. Trimethylamine N-Oxide, derived from gut microbial metabolism of dietary compounds, is linked to cardiovascular risks, inducing pro-inflammatory cytokines. Within obesity, adipose tissue macrophages secrete TNF-α [125], establishing a connection with diverse gut microbial species. As demonstrated in one study, individuals exhibiting an elevated abundance of *Bifidobacterium adolescentis* displayed reduced TNF-α production [126].

While these proposed mechanisms offer insights, establishing a comprehensive understanding requires further research to decipher the nuanced interplay between the gut microbiota and inflammatory processes.

Some studies have investigated the different mechanisms through which BPMs from EOs act on inflammation. Four mechanisms have been identified: the maintenance of the epithelial barrier, the reduction in inflammatory signaling pathways, the activation of sensory receptors, and the modulation of the microbiome [68].

The essential oil extracted from *C. aurantium*, with its main BPM being linalool, was found to suppress pro-inflammatory cytokine secretion (IL-6, TNF-α, and IL-1β), NO production, and COX-2 expression, as demonstrated in an in vitro model utilizing LPS-stimulated RAW264.7 macrophages [127]. Several studies have demonstrated the bioactive potential of menthol for regulating inflammation in animal models of colitis [128] and cystitis [129], by inhibiting the synthesis/release of inflammatory mediators such as IL-1β, COX-2, PGE2, and TNF-α.

In an in vivo study investigating the anti-inflammatory properties of BPMs in the composition of thyme (*Thymus magnus*) EO, researchers found that intraperitoneal administration of the EO exhibited a concentration-dependent analgesic effect, with the highest dosage showing superior pain relief compared to aspirin. Additionally, the study revealed that thyme EO suppressed ear edema in a concentration-dependent manner. These findings suggest that thyme BPMs exhibit anti-inflammatory activity, although further research is needed to fully understand its mechanisms before clinical application [130].

The modulation of the microbiome as a mechanism pertaining to the overall anti-inflammatory effects of EOs is an interesting aspect to be considered from the perspective of ALS management, since microbiome-related therapy could potentially be one of the possible areas of action. Some BPMs in the composition of Sichuan pepper (*Zanthoxylum bungeanum*) EO (ZBEO) have the potential to reduce myeloperoxidase (MPO) activity and levels of pro-inflammatory cytokines (TNF-α, IL-1β, and IL-12) in an animal model. This anti-inflammatory effect is crucial for regulating colonic inflammation, as pro-inflammatory cytokines play a pivotal role in inflammatory bowel disease, promoting oxidative stress. ZBEO treatment also restored the expression of zonulin, a tight junction protein essential for maintaining intestinal mucosal integrity and preventing bacterial infiltration. Moreover, ZBEO changed the composition of gut microbiota, reducing *E. coli* and increasing *Lactobacillus* spp. and *Bifidobacterium* spp. levels. These findings suggest that ZBEO could be valuable for therapeutic purposes in situations where dysbiosis, characterized by an increase in inflammatory markers, is present due to its potential to balance the microbiota and reduce gut inflammation [131].

Other studies, looking at thymol, a natural monoterpene phenolic BPM found in essential oils of *Lippia* and *Thymus* genera plants, have explored its potential to mitigate LPS-induced inflammation in IPEC-J2 cells by reducing ROS production and decreasing the overall levels of pro-inflammatory cytokines. The antioxidant effect of thymol, as well as its ability to modulate inflammatory signaling pathways, such as the MAPK/NFκB pathway, contributes to the decrease in the inflammatory response and the promotion of cellular homeostasis. In addition, thymol preserves epithelial barrier integrity [132], making it a strong candidate for fighting both inflammation and oxidative stress in pathologies where these mechanisms are present, such as neurodegenerative diseases and ALS.

**Figure 2:**
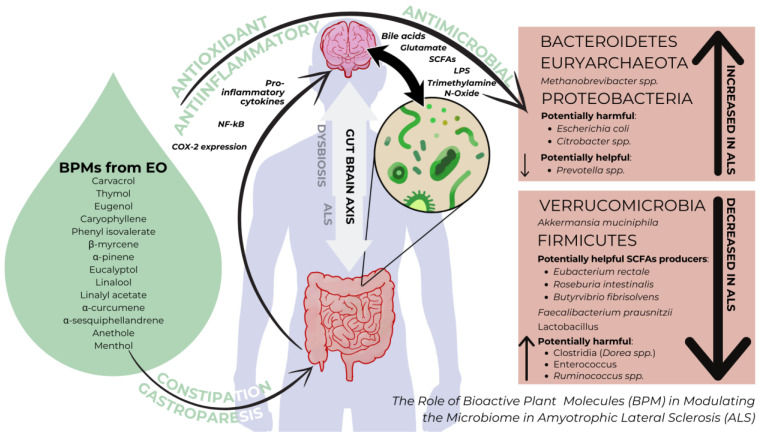
Gut microbiota imbalances in ALS and the proposed mechanism of action of BPMs from EO on the gut–brain axis.

The gut–brain axis, as illustrated in Figure 2, represents a complex two-way communication system linking the gut and the brain, where signals from the gut’s microbiome play a significant role alongside traditional brain-to-gut communication. This complex network, regulated by neural, endocrine, and immune pathways, presents an opportunity for modulation through plant bioactive molecules (BPMs). BPMs have the potential to directly affect inflammation, oxidative stress, and microbial balance, making them promising additions to the treatment toolkit for ALS. By harnessing the effects of BPMs with enteric action, we can explore a novel approach to address the multifaceted challenges of ALS, offering targeted interventions and potentially improving patient outcomes.

## 5. Conclusions

In conclusion, the potential of targeted bioactive plant molecules (BPMs) in ALS therapy lies in their ability to influence gut microbiome function. However, further research is essential to draw definitive conclusions. A deeper understanding of the microbiome’s role in ALS and the effects of BPMs necessitates consistent methods for determining microbiome–metabolome interactions. The efficacy of BPMs, particularly those derived from essential oils (EOs), depends on various factors such as their optimal dosage, absorption timing, and specific impact on gut microbiota. Integrating BPMs into tailored dietary interventions to address clinical gastrointestinal alterations in ALS is crucial. Yet, understanding the diverse effects of BPMs on gut microbiota in vivo remains pivotal for developing effective strategies against enteric infections, inflammation, and colonic dysbiosis.

## Data Availability

Data are unavailable due to privacy.
